# Enhancing the Mechanical Performance of Laser Powder Bed Fusion Prepared 316L Stainless Steel by Deformation Post-Processing at Ambient Temperature

**DOI:** 10.3390/ma19030615

**Published:** 2026-02-05

**Authors:** Radim Kocich, Lenka Kunčická

**Affiliations:** Department of Metallurgical Technologies, Faculty of Materials Science and Technology, VŠB Technical University of Ostrava, 17. listopadu 2172-15, 708 00 Ostrava, Czech Republic; radim.kocich@vsb.cz

**Keywords:** 316L stainless steel, laser powder bed fusion, rotary swaging, microstructure, mechanical properties

## Abstract

Preparation of metallic materials via laser powder bed fusion has gained high popularity primarily due to the versatility of the processed materials and the complexity of the available component geometries. However, the prepared components feature characteristic shortcomings. Among the ways to successfully reduce/eliminate printing issues and homogenize the properties within additively prepared materials is optimized post-processing. In this study, we present the positive effects of deformation post-processing at ambient (room) temperature on the microstructure and mechanical properties of AISI 316L stainless steel prepared by laser powder bed fusion. The post-processing was performed by the industrially applicable method of rotary swaging, for which varying swaging degrees were applied. The selected swaging degree influenced primarily the interactions between the dynamic strengthening and softening processes and consequently the strength/plasticity ratio, although all the applied swaging degrees successfully eliminated the residual porosity and imparted (sub)structure development and grain refinement. The ultimate tensile strength (UTS) for the original workpiece was 282 MPa, and it increased up to more than 1400 MPa after the final swaging while maintaining favorable plasticity (elongation to failure over 30%). The study thus proposes a way to successfully enhance the performance of additively manufactured AISI 316L steel with the use of a commercially applicable plastic deformation technology.

## 1. Introduction

Among the most commonly used methods of additive manufacturing are, for example, Selective Laser Melting (SLM) [1,2], Selective Laser Sintering (SLS) [3,4], Direct Metal Laser Sintering (DMLS) [5,6], Direct Energy Deposition (DED) [7,8], Direct Metal Deposition (DMD) [9,10], and more. Laser powder bed fusion (PBF-LB/M) [11,12] processes of additive manufacturing have gained popularity primarily for the possibility of (i) manufacturing components of more or less complex geometries, and (ii) fabricating components featuring intriguing combinations of materials (such as composites [13,14], functionally gradient materials [15,16], or materials with dispersions of fine oxides [17]). Although numerous methods of additive manufacturing have demonstrated remarkable success in the fabrication of specific components [18,19], PBF-LB/M processes still exhibit certain issues. Among these are the occurrence of residual porosity and inhomogeneous distribution of residual stress within the fabricated materials and components, their surface roughness, and possible shape distortions [20,21,22].

The internal structures and thus properties of PBF-LB/M materials and components can be improved by optimized post-processing. Such post-processing typically involves heat treatment [23,24], although a sole heat treatment is not able to provide satisfactory elimination of the residual porosity. From the viewpoint of elimination of internal voids, a combination of heat treatment with high pressure, i.e., the hot isostatic pressing (HIP) method, is more effective [25,26]. Another approach is to apply post-processing via deformation or thermomechanical treatments [27,28,29]. Such treatments have been reported to advantageously eliminate residual porosity [30], as well as to improve the overall performance of PBF-LB/M-fabricated materials [31,32]. For the purposes of deformation or thermomechanical post-processing treatments, a variety of conventional, as well as non-conventional, techniques can be used. Among the conventional ones are, for example, die forging [33,34], rolling [35,36], and extrusion [37,38]. The unconventional ones involve methods of severe plastic deformation (SPD), e.g., equal channel angular pressing (ECAP) [39,40] and various ECAP modifications (ECAP with (partial) back pressure [41,42], ECAP-Conform [43,44], twist channel angular pressing, TCAP [45,46,47], twist channel multi-angular pressing TCMAP [48,49], etc.), and high-pressure torsion (HPT) [50,51], as well as methods of intensive plastic deformation (IPD), such as multi-axial forging [52,53] and rotary swaging (RS) [54,55].

RS is a manufacturing process that is used commercially, especially in the automotive, aerospace, and aviation industries. It is applicable under a variety of temperature conditions [56,57]. During the process, gradual strain increments are introduced into the swaged piece. Moreover, compressive and rotational forces affect the swaged piece directly, and swaging thus promotes consolidation and shear mixing. Therefore, RS can advantageously be used for tailored thermomechanical (post-)processing to directly dynamically control the development of porosity, stress state, and microstructure, and consequently to affect the final properties [58,59]. Besides conventionally prepared bulk alloys and compounds [60,61], RS is suitable for the processing of challenging materials, such as composites [62,63], laminates [64,65], gradient structures [66,67], or powder-based materials [68,69].

AISI 316L stainless steel features highly favorable mechanical and utility properties. It is commonly used in a variety of commercial and industrial applications in the (nuclear) energetics [70,71], transportation, marine [72,73], and (petro)chemical industries [74,75], medicine [76,77], and more. The microstructure of an austenitic stainless steel, ultimately affecting its properties and behavior, generally depends on its chemical composition and the content (ratio) of the ferrite and austenite stabilizing elements, especially chromium and nickel [78]. While the primary role of nickel is to stabilize the austenite phase and provide the steel with enhanced heat resistance, chromium primarily improves corrosion resistance [79]. Nevertheless, the microstructure is also affected by the (post-)processing parameters, especially the temperature. The commonly used stainless steel components are typically manufactured by (the above-mentioned) conventional processing methods. However, when subjected to processing under warm conditions, typically at temperatures between 500 °C and 800 °C, numerous phase changes can occur [80,81,82]. Among these is sensitization, i.e., segregation of chromium carbides, as a result of which chromium content can be locally depleted in certain regions of a steel component (especially for steels with high carbon contents), which can lead to the development of localized corrosion. Therefore, processing at ambient temperatures seems to be highly favorable from the viewpoint of tailoring the microstructure development and avoiding undesired phase changes and segregations.

With the progress and developments in modern industrial fields, numerous specific applications have been introduced in which the components are exposed to challenging conditions and thus need to exhibit defined microstructures and exceptional performance. For this reason, researchers are working continuously not only on the development of new processing methods and the optimization of the developed production processes, but also on the characterization of their effects on the microstructures and properties of the stainless steel to introduce new features and further enhance its performance [83,84,85]. Additive manufacturing has also been applied to fabricate AISI 316L stainless steel components, although the history of this research topic is still relatively short. Researchers have investigated the development of microstructure, primarily in relation to the applied types of laser energy delivery [86,87,88] or the introduced mechanical properties [83,89,90]. Others have also focused on the correlations of microstructure and other material properties, such as residual stress [91], fatigue [74,92], or machinability [93]. The possibilities of surface enhancement and thus reduction/elimination of the as-printed surface roughness have also been investigated [94,95]. Nevertheless, the effects of deformation and thermomechanical post-processing executed via industrially applicable techniques on the microstructures and performance of PBF-LB/M fabricated AISI 316L steel have not been sufficiently investigated yet.

The primary focus of the herein-presented research was to investigate the effects of deformation post-processing of PBF-LB/M-fabricated 316L stainless steel on its microstructure development and mechanical properties. Given the ever-increasing interest in the development of innovative production methods, the initial workpieces were fabricated by the popular SLM additive manufacturing technology. The post-processing was then carried out via the commercially applicable RS, which was executed at ambient (room) temperature. The processing temperature was selected with the intention of (i) saving time and production costs by eliminating the need for heating, and (ii) designing and introducing a stabilized microstructure. In order to monitor the progress of structure-forming processes during the deformation post-processing and its effects on the development of the mechanical properties, several swaging degrees were evaluated.

## 2. Materials and Methods

### 2.1. Preparation of Experimental Material

The original AISI 316L stainless steel workpieces used for this study were manufactured by PBF-LB/M (SLM) under an inert Ar atmosphere with a purity of 99.998% using an AM400 3D-printing machine (Renishaw plc., Wotton-under-Edge, UK). The original 316L stainless steel (DIN 1.4404) powder featured a particle size distribution between 15 and 45 µm (both 3D printer and original powder by Renishaw plc., Wotton-under-Edge, UK). Detailed characteristics of the powder can be found in the relevant datasheet by Renishaw, plc. The chemical composition of the powder, as declared by the producer (Renishaw plc.) and measured herein by optical emission spectroscopy (SPECTROMAXx device by SPECTRO Analytical Instruments, Kleve, Germany), is depicted in Table 1.

The initial PBF-LB/M-fabricated workpieces were designed to be 100 mm in length and 25 mm in diameter. As regards the effects of printing parameters and strategy, the following set-up was used based on our previous experience with PBF-LB/M-fabricated materials (e.g., [30]): meander type vertical printing strategy (respective to the largest dimension of the PBF-LB/M workpiece), 50 µm printing layer thickness, 200 W hatch laser power, 80 µs hatch exposure time, 60 µm hatch point distance, 50 µm hatch spacing, 650 mm·s^−1^ scan speed, and 70 µm focus size. The PBF-LB/M-fabricated workpieces were subsequently subjected to 950 °C/30 min heat treatment to introduce (partial) homogenization and relaxation of the residual stress. The heat treatment temperature of 950 °C was selected having in mind two opposite criteria: the selected temperature should be the lowest possible to prevent undesired grain growth and, simultaneously, high enough to prevent sensitization [96,97]. Similarly, the dwell time was rather short to reduce possible negative effects on the microstructure (grain growth) and, therefore, the utility properties [98,99]. This initial material state is further denoted as L-PBF sample.

The PBF-LB/M workpieces were subsequently subjected to deformation post-processing via RS at ambient (room) temperature. The diameter of the PBF-LB/M workpiece was gradually reduced to diameters of 20 mm (sample 20), 17.5 mm (sample 17.5.), and finally 15 mm (sample 15). The swaging degrees for the swaged pieces were calculated using Equation (1):

(1) $$\varphi =\frac{S_{0}}{S_{n}},$$

where *S*_0_ and *S*_n_ are the cross-sectional areas of the swaged piece at input and output from swaging dies, respectively. The swaging degree for sample 20 was 0.45, for sample 17.5 it was 0.7, and for sample 15 it was 1.0.

### 2.2. Analyses

#### 2.2.1. Microstructure

The analyses of the microstructures of the original PBF-LB/M workpiece and the swaged pieces were performed via scanning electron microscopy (SEM), particularly the electron backscatter diffraction (EBSD) method. The samples for EBSD scanning were prepared from cross-sectional cuts through the pieces by manual grinding on SiC papers to a coarseness of 1200, and subsequent manual polishing using 3 µm and 1 µm polycrystallic diamond solutions (equipment and accessories by QATM, delivered by Metalco Testing s.r.o., Roztoky u Prahy, Czech Republic). The microscopy observations on the cross-sectionally prepared samples were performed in a half-radius distance for each sample diameter. The EBSD analysis for the L-PBF sample was performed on an area of 500 × 400 µm^2^, with a 1.0 µm scan step, as the grains were expected to be the coarsest there. For the swaged pieces, the EBSD observations were also performed with higher precision to observe the microstructures in greater detail. The detailed scanned areas for these samples were 30 × 30 µm^2^ and the applied scan step was 0.075 µm. For the evaluations of the grains, the considered grain boundary limits were 5° for low-angle grain boundaries (LAGBs), and 15° for high-angle grain boundaries (HAGBs). The ideal texture orientations were evaluated with a maximum deviation of 15°. The EBSD data were processed using ATEX (Analysis Tools for Electron and X-ray diffraction) software (version 5.12, Université de Lorraine, Metz, France) [100].

#### 2.2.2. Mechanical Properties

The mechanical properties of the PBF-LB/M workpiece and the swaged pieces were investigated via Vickers microhardness measurements and using tensile tests. Vickers microhardness measurements were executed with a load of 1000 g (HV1) and a load time of 10 s for each indent (Zwick Roell DuraScan 70 G5 device by Zwick Roell CZ s.r.o., Brno, Czech Republic). The values were measured across the entire cross-sections of the prepared samples, with a spacing between the individual indents of 1 mm. The measured HV values were then used to create microhardness maps documenting the development of the mechanical properties within the workpiece during the entire swaging process.

**Figure 1:**
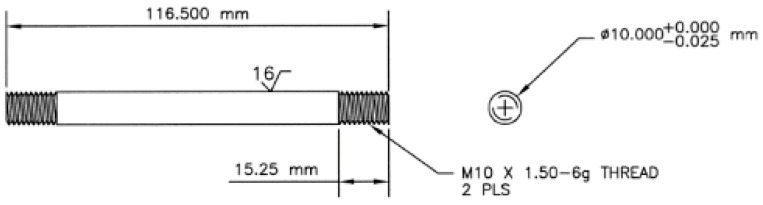
Schematic drawing of tensile test sample.

The tensile tests were performed at a strain rate of 10^−3^ s^−1^ using the versatile Gleeble 3800 plastometer (by Dynamic Systems Inc., Poestenkill, NY, USA). For each material state, four individual samples were prepared and subsequently tested to provide data for calculating the average values for each material state. The shape and dimensions of the cylindrical testing samples are depicted in the schematic drawing in Figure 1.

## 3. Results and Discussion

### 3.1. Microstructures

#### 3.1.1. L-PBF Sample

**Figure 2:**
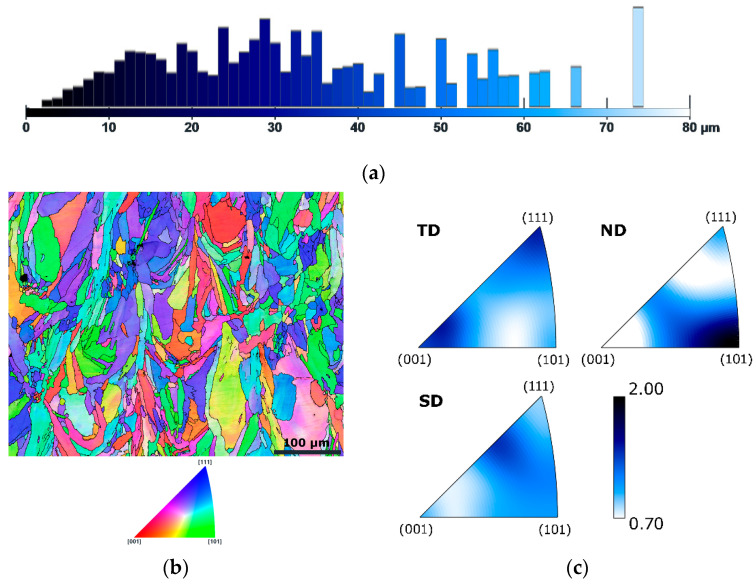
Results of microstructure analysis for L-PBF sample: graphical depiction of area-weighted grain sizes (**a**); OIM image (**b**), IPF texture plots (**c**).

At first, the microstructure of the original workpiece was examined. Figure 2a shows the graphical depiction of area-weighted grain size fractions for the L-PBF sample. As can be seen, the majority of the grains within the L-PBF sample had sizes between 10 and 40 µm, although the microstructure also featured larger grains with sizes exceeding 70 µm. The average grain size for the L-PBF sample was 36.2 µm. The Orientation Image Map (OIM), depicted in Figure 2b, shows that the as-printed grains featured a typical melt pool character. The figure also reveals the evident presence of pores within the as-printed microstructure. The grains did not feature any prevailing preferential orientation, which can be noted from the OIM image in Figure 2b, but this was also confirmed by the Inverse Pole Figure (IPF) texture plots depicted in Figure 2c. The maximum texture intensity for the L-PBF sample was less than two times random. The herein-acquired findings correspond to typical structures of PBF-LB/M-fabricated materials (see e.g., [101,102,103]).

#### 3.1.2. Swaged Pieces

**Figure 3:**
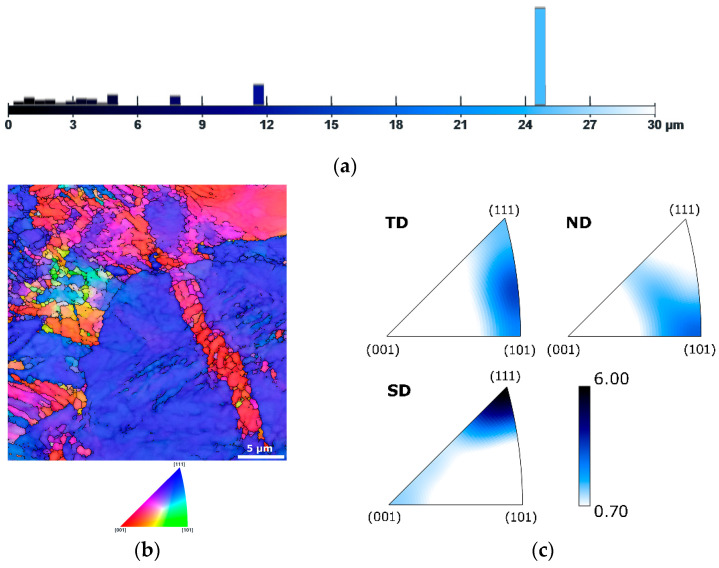
Results of microstructure analysis for sample 20: graphical depiction of area-weighted grain sizes (**a**); OIM image (**b**), IPF texture plots (**c**).

Figure 3a shows a graphical depiction of the area-weighted grain size fractions for sample 20, while Figure 3b shows the OIM image and Figure 3c depicts the IPF texture plots for sample 20, subjected to a swaging degree of 0.45. As shown in Figure 3a, the grain sizes decreased as a result of the effect of swaging. However, the grain size was inhomogeneous and the microstructure still featured relatively large grains. Nevertheless, numerous grains had already been refined by the deformation post-processing even with this relatively low swaging ratio, as documented by the randomly oriented fine grains in the OIM image in Figure 3b. Swaging with a degree of 0.45 promoted fiber texture formation. The IPF texture plots in Figure 3c show that the grains exhibited the (001)||SD and (111)||SD preferential texture orientations, and the maximum texture intensity reached 6.0. Also notable was the absence of voids and pores within the deformed microstructure. Swaging at ambient temperature with a swaging degree of 0.45 was thus sufficient to partially refine the grains and eliminate voids within the original L-PBF steel sample.

**Figure 4:**
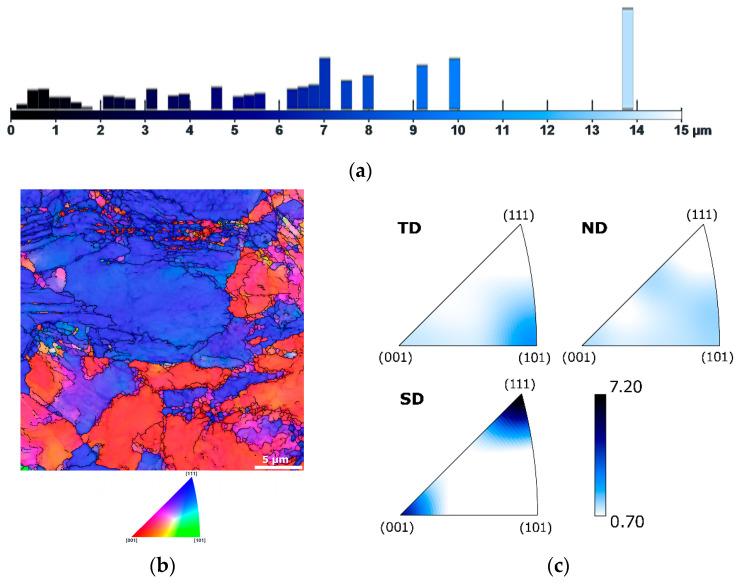
Results of microstructure analysis for sample 17.5: graphical depiction of area-weighted grain sizes (**a**); OIM image (**b**), IPF texture plots (**c**).

Increasing the swaging degree further contributed to grain size refinement and microstructure homogenization. The sizes of the largest grains within the microstructure of sample 17.5 reached up to 15 µm. However, the microstructure also featured a considerable portion of grains smaller than 2 µm; see Figure 4a, which shows the respective area-weighted grain size graphical depiction. Figure 4b shows the OIM image for the microstructure of the sample subjected to a swaging degree of 0.7. The OIM confirmed that the microstructure featured a mix of refined and relatively larger grains, with a tendency to form the (001)||SD and (111)||SD preferential texture fibers (see also Figure 4c, depicting the IPF texture plots for sample 17.5). Numerous grains also featured a developed substructure, depicted as smaller (sub)grains with different orientations in Figure 4b. Some of the larger grains even exhibited traces of shear bands. Such a (sub)structure development points to the simultaneous occurrence of dynamic strengthening and softening processes during swaging [104]; this hypothesis will further be discussed in relation to mechanical properties in Section 3.2.

**Figure 5:**
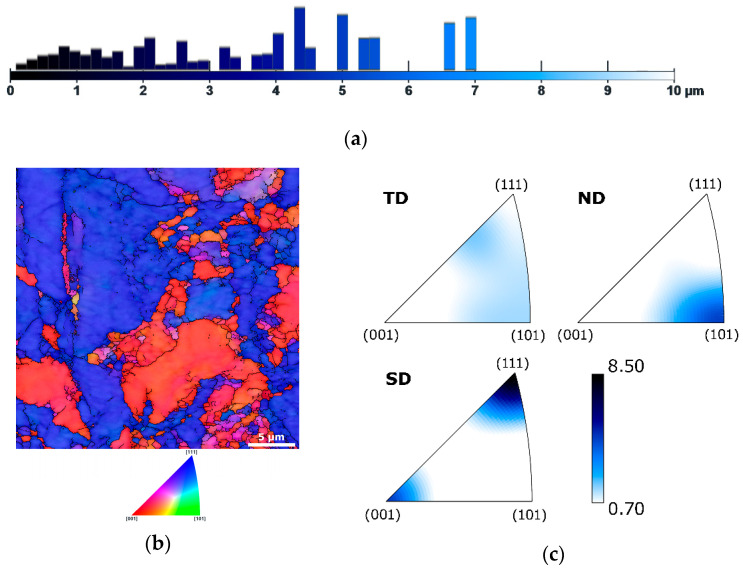
Results of microstructure analysis for sample 15: graphical depiction of area-weighted grain sizes (**a**); OIM image (**b**), IPF texture plots (**c**).

Last but not least, the microstructure of sample 15 was examined. Figure 5a shows the graphical depiction of area-weighted grain size fractions for sample 15, Figure 5b depicts the OIM image for this sample, and Figure 5c shows the IPF texture plots for the sample subjected to a swaging degree of 1.0. The figures document that further increase in the swaging degree resulted in further grain size refinement. The (partially) occurring dynamic restoration meant that sample 15 still featured relatively larger grains with sizes of up to 7 µm. However, the microstructure consisted of a majority of refined grains with very fine, even ultrafine (UF, smaller than 1 µm), grain sizes (see also the OIM in Figure 5b). The average grain size for this sample decreased to 4.6 µm. The gradual room temperature swaging also introduced the formation of relatively intense (001)||SD and (111)||SD fiber textures. The maximum texture intensity for sample 15 reached 8.5; see the IPF texture plots in Figure 5c. The increased maximum texture intensity, when compared to sample 17.5, gives rise to a supposition that dynamic strengthening, i.e., the development of dislocations substructure, dominated over dynamic softening, i.e., the annihilation of dislocations (dynamic recovery) and/or the formation of new relaxed grains (dynamic recrystallization), during swaging to a final diameter of 15 mm [105]. This hypothesis is further discussed below in Section 3.2. on mechanical properties.

### 3.2. Mechanical Properties

#### 3.2.1. Vickers Microhardness

**Figure 6:**
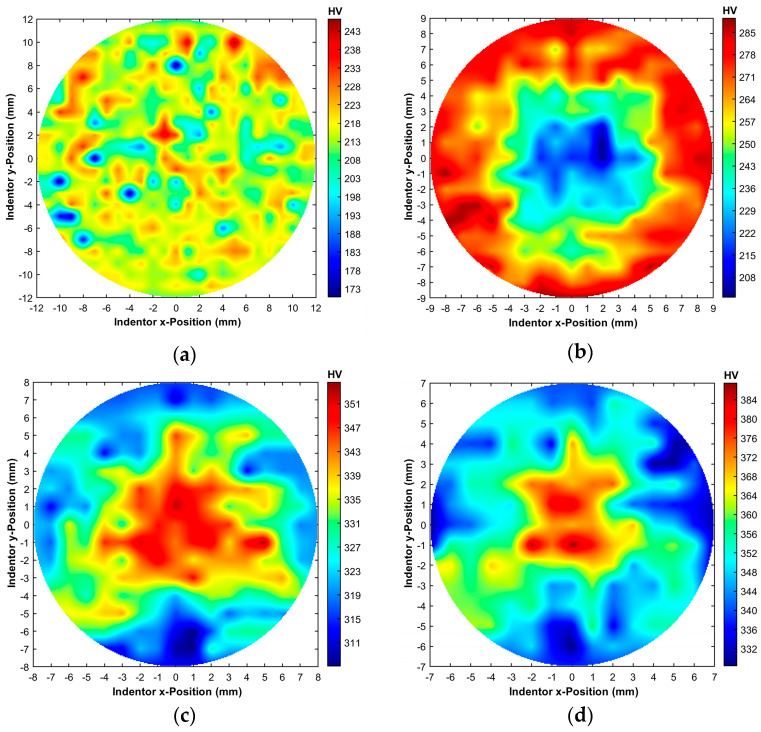
Maps of HV1 Vickers microhardness values acquired across cross-sections of investigated samples: L-PBF sample (**a**); sample 20 (**b**); sample 17.5 (**c**); sample 15 (**d**).

The results of Vickers microhardness measurements for the L-PBF sample and the swaged samples are graphically depicted in Figure 6a–d as maps of the HV1 microhardness values acquired across the cross-sections of the samples. The average HV1 value for the original workpiece was 215 HV1. However, the L-PBF sample exhibited substantial cross-sectional microhardness inhomogeneity, as can be seen in Figure 6a. The local drops in the HV1 values observed for this sample can most probably be attributed to the presence of voids and printing defects, as was depicted also in the OIM image in Figure 2b. Processed with a swaging degree of 0.45, sample 20 exhibited an increased average microhardness of 259 HV1. Nevertheless, the inhomogeneity of the HV1 values acquired across the cross-section of this sample was the highest observed (standard deviation of 19.8); see Figure 6b. This phenomenon can be explained by taking into consideration the relatively low applied swaging degree, together with the character of the swaging process. During swaging, the swaging dies rotating around the swaged piece gradually introduce small strain increments from the peripheral region of the swaged piece towards its axial region (see e.g., [56,57]). The peripheral region of the swaged piece is thus influenced by the imposed shear strain from the very beginning of swaging. Consequently, for relatively low swaging degrees, the imposed strain is accumulated primarily at the outer cross-sectional region of a swaged piece. The effect of the imposed strain then gradually penetrates towards its axial region with increasing swaging degree. For this reason, the periphery (outer cross-sectional region) of sample 20 exhibited increased microhardness compared to the L-PBF sample, while the axial region of sample 20 was still only mildly affected by the applied deformation. Consequently, sample 20 exhibited high cross-sectional inhomogeneity of HV1 values. This hypothesis corresponds to the above-presented results of microstructure observations (Figure 3b).

Increasing the swaging degree to 0.7, and finally to 1.0, then introduced structure changes also in the axial region of the swaged piece. Sample 17.5 (Figure 6c) featured an average microhardness of 330 HV1, and for sample 15 (Figure 6d), the average microhardness increased to 352 HV1. Although the values for both of the samples increased when compared to the L-PBF sample and sample 20, which corresponds to the above-mentioned microstructure observations and hypothesis on deformation strengthening introduced by swaging (see also the discussion in Section 3.2.2. on tensile testing), sample 17.5 and sample 15 both exhibited different trends in microhardness distributions across their cross-sections compared to sample 20. In other words, sample 17.5 and sample 15 both exhibited higher HV1 values in the axial region than along the periphery. The increase in microhardness in the axial regions of the samples corresponds to the above-mentioned trend of imposing the strain during swaging. However, it also gives rise to a supposition of (partial) development of dynamic restoration processes in the peripheral regions of the samples. In order to confirm this hypothesis, the results of Vickers microhardness mapping have to be related to data on the stress–strain behaviors of the prepared pieces.

#### 3.2.2. Stress–Strain Data

**Figure 7:**
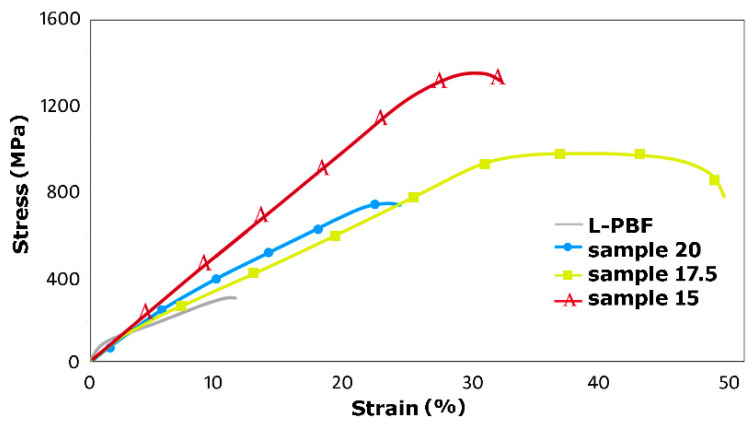
Tensile-tests-based stress–strain data.

Figure 7 depicts the average stress–strain curves acquired during tensile testing of the L-PBF and the swaged samples. The tensile tests results support the results of microhardness measurements and document that the swaging process enhanced the mechanical properties (both plasticity and strength) of the L-PBF sample, regardless of the applied swaging degree. However, the different swaging degrees introduced differences in the behaviors of the individual swaged pieces. The L-PBF sample exhibited an ultimate tensile strength, UTS, of 282 MPa and relatively poor plasticity (elongation to failure of 11.8%). This behavior can primarily be attributed to the presence of printing defects and cross-sectional inhomogeneity, as also documented by the microhardness map in Figure 6a. Sample 20, processed with the lowest swaging degree, then exhibited an increase in both strength and plasticity. The respective stress–strain curve featured a relatively rapid increase in strength with increasing imposed strain. However, the plasticity for sample 20 was the lowest among the tested swaged samples. The most probable reason for such a behavior was the relatively inhomogeneous microstructure of this sample, which is in accordance with the above-presented results of microstructure observations (sample 20 exhibited inhomogeneous microstructure consisting of a mix of refined and coarse grains; see Figure 2b), and Vickers microhardness measurements (Figure 6b showed that sample 20 featured inhomogeneities and that the axial region of the sample was affected only mildly by the swaging).

Increasing the swaging degree then positively affected the mechanical properties of the L-PBF sample. Sample 17.5 exhibited a less steep increase in strength during testing, i.e., the deformation hardening for this sample was not as rapid as for sample 20. Also, this sample exhibited an increase in the UTS (compare 1031 MPa for sample 17.5 to 776 MPa acquired for sample 20) and remarkably higher elongation to failure (compare 48.6% for sample 17.5 to 22.4% acquired for sample 20). Also notable was the intriguing course of the stress–strain curve, particularly after the yield strength was reached. The curve exhibited a characteristic plateau in the strain region of approximately 30–40%; none of the tested samples exhibited such a behavior. This phenomenon can be attributed to the achievement of a balance state between the dynamic structure-forming processes of strengthening and softening [106]. Swaging with a swaging degree of 0.7 thus, most probably, resulted in accumulation of the imposed energy within the swaged piece to an extent sufficient to activate dynamic recovery [105]. This behavior, i.e., partial balance between dynamic strengthening and softening [107], thus provided sample 17.5 with the highest plasticity (elongation to failure) of all the examined samples.

Sample 15, subjected to the highest swaging degree, then again exhibited a different behavior. The particular conditions selected for the swaging process, i.e., especially processing temperature and swaging degree, were documented to affect (remarkably) not only the microstructures but also the mechanical and utility properties of the swaged pieces, as the selected conditions primarily affected the material plastic flow and distribution of the imposed strain across their bulk volumes (e.g., [62,108,109], see also the above-mentioned results of Vickers microhardness measurements). In other words, the interactions of the compressive force with the radial force and shear strain affecting particular regions along the swaged piece can impart particular microstructure changes, such as the accumulation of high densities of dislocations [110] or the formation of deformation induced martensite [111,112], all of which can contribute to a substantially high material strength (UTS). Sample 15 exhibited the steepest slope of the stress–strain curve, i.e., the highest deformation strengthening rate, among the tested samples. However, the plasticity, i.e., elongation to failure, for this sample decreased compared to sample 17.5 (but was still higher when compared to sample 20). Increasing the swaging degree to 1.0 thus again supported the development of dynamic strengthening at the expense of dynamic softening, which resulted in the observed decrease in the plastic properties for this sample, together with an increase in the UTS up to 1412 MPa.

## 4. Conclusions

This study presented the effects of post-processing of AISI 316L stainless steel, prepared by selected laser melting, by gradual rotary swaging at ambient (room) temperature on the microstructure and mechanical properties of the steel workpieces. The results showed that the swaging degree had a primary influence on the interactions of dynamic strengthening and softening processes. The selected post-processing method is suitable to be used to enhance the properties of the additively manufactured steel based on the following findings:

- all the post-processing regimes successfully eliminated residual porosity and imparted (sub)structure development and grain refinement, positively affecting the final mechanical properties;
- even the lowest selected swaging degree of 0.45 improved the mechanical properties, and the UTS increased from 282 MPa (original as-printed workpiece) to 776 MPa;
- increasing the swaging degree to 0.7 introduced (partial) balance between dynamic strengthening and softening; the swaged piece featured a UTS of 1031 MPa, together with highly enhanced plasticity (elongation to failure of 48.6%);
- the highest swaging degree of 1.0 introduced a homogeneous microstructure, with grains refined to an average size of less than 5 µm, and the highest UTS exceeding 1400 MPa.

The results of this study document that deformation post-processing at ambient temperature with optimized processing conditions can be used not only to successfully eliminate printing defects within laser powder bed fusion prepared stainless steel, but also to control the development of dynamic structure-forming processes and to tailor the final properties.

## Figures and Tables

**Table 1:** Chemical composition of initial AISI 316L stainless steel powder, as declared by Renishaw, plc., and measured. Renishaw plc., Data sheets—Additive manufacturing. Available online: https://www.renishaw.com/en/data-sheets-additive-manufacturing--42225 (accessed on 15 June 2025).

Element	Cr	Ni	Mo	Mn	Si	O	N	C	S	Fe
As declared [wt.%]	16–18	10–14	2–3	≤2	≤1	≤0.1	≤0.1	≤0.03	≤0.03	bal.
As measured [wt.%]	18.1 ± 0.3	10.9 ± 1.0	2.1 ± 0.2	1.7 ± 0.3	1.0 ± 0.1	0.06 ± 0.02	0.02 ± 0.01	0.02 ± 0.01	0.003 ± 0.002	bal.

## Data Availability

The original contributions presented in the study are included in the article, further inquiries can be directed to the corresponding author.

## References

[1] Yadroitsev I., Krakhmalev P., Yadroitsava I. (2014). Selective Laser Melting of Ti6Al4V Alloy for Biomedical Applications: Temperature Monitoring and Microstructural Evolution. J. Alloys Compd..

[2] Ratkevich G.V., Zhdanov A.V., Belyaev L.V., Yugov V.I., Afanas’eva L.E. (2019). Selective Laser Melting of Corrosion-Resistant Steel. Russ. Metall. (Met.).

[3] Shishkovsky I., Yadroitsev I., Bertrand P.H., Smurov I. (2007). Alumina–Zirconium Ceramics Synthesis by Selective Laser Sintering/Melting. Appl. Surf. Sci..

[4] Antonov E.N., Dunaev A.G., Konovalov A.N., Minaeva S.A., Popov V.K. (2020). Temperature Field Distribution in Polymer Particles during Surface-Selective Laser Sintering. Laser Phys..

[5] Kazantseva N., Krakhmalev P., Yadroitsev I., Fefelov A., Merkushev A., Ilyinikh M., Vinogradova N., Ezhov I., Kurennykh T. (2017). Oxygen and Nitrogen Concentrations in the Ti-6Al-4V Alloy Manufactured by Direct Metal Laser Sintering (DMLS) Process. Mater. Lett..

[6] Barucca G., Santecchia E., Majni G., Girardin E., Bassoli E., Denti L., Gatto A., Iuliano L., Moskalewicz T., Mengucci P. (2015). Structural Characterization of Biomedical Co–Cr–Mo Components Produced by Direct Metal Laser Sintering. Mater. Sci. Eng. C.

[7] Shishkovsky I., Kakovkina N., Nosova E., Khaimovich A. (2023). Laser In Situ Synthesis of Gradient Fe-Ti Composite during Direct Energy Deposition Process. J. Manuf. Mater. Process..

[8] Dmitrieva A., Mukin D., Sorokin I., Stankevich S., Klimova-Korsmik O. (2023). Laser-Directed Energy Deposition of Ni-Based Superalloys with a High Content of γ′-Phase Using Induction Heating. Mater. Lett..

[9] Dalaee M.T., Gloor L., Leinenbach C., Wegener K. (2020). Experimental and Numerical Study of the Influence of Induction Heating Process on Build Rates Induction Heating-Assisted Laser Direct Metal Deposition (IH-DMD). Surf. Coat. Technol..

[10] Kupriyanova O.A., Gorlenko D.A., Sheksheev M.A., Pivovarova K.G., Ognyeva E.M., Polyakova M.A. (2023). Studying the Formation of the Microstructure of Powdered Steel with Trip Effect by Direct Metal Deposition. Metallurgist.

[11] Voloskov B., Mishurova T., Evlashin S., Akhatov I., Bruno G., Sergeichev I. (2023). Artificial Defects in 316L Stainless Steel Produced by Laser Powder Bed Fusion: Printability, Microstructure, and Effects on the Very-High-Cycle Fatigue Behavior. Adv. Eng. Mater..

[12] Nadammal N., Mishurova T., Fritsch T., Serrano-Munoz I., Kromm A., Haberland C., Portella P.D., Bruno G. (2021). Critical Role of Scan Strategies on the Development of Microstructure, Texture, and Residual Stresses during Laser Powder Bed Fusion Additive Manufacturing. Addit. Manuf..

[13] Tarasova T., Gvozdeva G., Ableyeva R. (2019). Aluminium Matrix Composites Produced by Laser Based Additive Manufacturing. Mater. Today Proc..

[14] Zykova A., Panfilov A., Chumaevskii A., Vorontsov A., Moskvichev E., Nikonov S., Gurianov D., Savchenko N., Kolubaev E., Tarasov S. (2023). In-Situ Dispersion Hardened Aluminum Bronze/Steel Composites Prepared Using a Double Wire Electron Beam Additive Manufacturing. Prog. Addit. Manuf..

[15] Kavousi Sisi A., Ozherelkov D., Chernyshikhin S., Pelevin I., Kharitonova N., Gromov A. (2024). Functionally Graded Multi-Materials by Laser Powder Bed Fusion: A Review on Experimental Studies. Prog. Addit. Manuf..

[16] Ghorbanpour S., Sahu S., Deshmukh K., Borisov E., Riemslag T., Reinton E., Bertolo V., Jiang Q., Popovich A., Shamshurin A. (2021). Effect of Microstructure Induced Anisotropy on Fatigue Behaviour of Functionally Graded Inconel 718 Fabricated by Additive Manufacturing. Mater. Charact..

[17] Wang Y., Wang B., Luo L., Oliveira J.P., Li B., Yan H., Liu T., Zhao J., Wang L., Su Y. (2023). Effects of Process Atmosphere on Additively Manufactured FeCrAl Oxide Dispersion Strengthened Steel: Printability, Microstructure and Tensile Properties. Mater. Sci. Eng. A.

[18] Kilina P., Drozdov A., Kuchumov A.G., Morozov E., Sirotenko L., Smetkin A. (2024). Two-Staged Technology for CoCr Stent Production by SLM. Materials.

[19] Kiselevskiy M.V., Anisimova N.Y., Kapustin A.V., Ryzhkin A.A., Kuznetsova D.N., Polyakova V.V., Enikeev N.A. (2023). Development of Bioactive Scaffolds for Orthopedic Applications by Designing Additively Manufactured Titanium Porous Structures: A Critical Review. Biomimetics.

[20] Chen Y., Peng X., Kong L., Dong G., Remani A., Leach R. (2021). Defect Inspection Technologies for Additive Manufacturing. Int. J. Extrem. Manuf..

[21] Sous F., Herrig T., Bergs T., Karges F., Feiling N., Zeis M. (2022). Electrochemical Defect Analysis of Additive Manufactured Components. J. Eng. Gas Turbine. Power.

[22] Brennan M.C., Keist J.S., Palmer T.A. (2021). Defects in Metal Additive Manufacturing Processes. J. Mater. Eng. Perform..

[23] Laleh M., Sadeghi E., Revilla R.I., Chao Q., Haghdadi N., Hughes A.E., Xu W., De Graeve I., Qian M., Gibson I. (2023). Heat Treatment for Metal Additive Manufacturing. Prog. Mater. Sci..

[24] Resnina N., Palani I.A., Belyaev S., Singh S., Liulchak P., Karaseva U., Mani Prabu S.S., Jayachandran S., Kalganov V., Iaparova E. (2021). Influence of Heat Treatment on the Structure and Martensitic Transformation in NiTi Alloy Produced by Wire Arc Additive Manufacturing. Materialia.

[25] Mclean N., Bermingham M.J., Colegrove P., Sales A., Soro N., Ng C.H., Dargusch M.S. (2022). Effect of Hot Isostatic Pressing and Heat Treatments on Porosity of Wire Arc Additive Manufactured Al 2319. J. Mater. Process. Technol..

[26] Petrovskiy P., Travyanov A., Cheverikin V.V., Chereshneva A.A., Sova A., Smurov I. (2020). Effect of Encapsulated Hot Isostatic Pressing on Properties of Ti6Al4V Deposits Produced by Cold Spray. Int. J. Adv. Manuf. Technol..

[27] Shakil S.I.I., Smith N.R.R., Yoder S.P.P., Ross B.E.E., Alvarado D.J.J., Hadadzadeh A., Haghshenas M. (2022). Post Fabrication Thermomechanical Processing of Additive Manufactured Metals: A Review. J. Manuf. Process..

[28] Peng X., Kong L., Fuh J.Y.H., Wang H. (2021). A Review of Post-Processing Technologies in Additive Manufacturing. J. Manuf. Mater. Process..

[29] Pourrahimi S., Hof L.A. (2024). On the Post-Processing of Complex Additive Manufactured Metallic Parts: A Review. Adv. Eng. Mater..

[30] Kunčická L., Kocich R., Németh G., Dvořák K., Pagáč M. (2022). Effect of Post Process Shear Straining on Structure and Mechanical Properties of 316 L Stainless Steel Manufactured via Powder Bed Fusion. Addit. Manuf..

[31] Mahajan A., Devgan S. (2025). Recent Advances in Surface Engineering of Additive Manufactured Materials for Enhancing Corrosion Resistance. Prog. Addit. Manuf..

[32] Kunčická L., Kocich R. (2024). High Strain Rate Induced Shear Banding within Additively Manufactured AISI 316L. Mater. Lett..

[33] Meiners F., Ihne J., Jürgens P., Hemes S., Mathes M., Sizova I., Bambach M., Hama-Saleh R., Weisheit A. (2020). New Hybrid Manufacturing Routes Combining Forging and Additive Manufacturing to Efficiently Produce High Performance Components from Ti-6Al-4V. Procedia Manuf..

[34] Ghassemali E., Tan M.J., Wah C.B., Jarfors A.E.W., Lim S.C.V. (2013). Grain Size and Workpiece Dimension Effects on Material Flow in an Open-Die Micro-Forging/Extrusion Process. Mater. Sci. Eng. A.

[35] Lukáč P., Kocich R., Greger M., Padalka O., Szaraz Z. (2007). Microstructure of AZ31 and AZ61 Mg Alloys Prepared by Rolling and ECAP. Kov. Mater. Met. Mater..

[36] Pustovoytov D., Pesin A., Tandon P. (2021). Asymmetric (Hot, Warm, Cold, Cryo) Rolling of Light Alloys: A Review. Metals.

[37] Sharma S., Mudgal D., Gupta V. (2024). Integrating Extrusion Process and Additive Manufacturing for Biomedical Breakthroughs. Int. J. Interact. Des. Manuf. (IJIDeM).

[38] Guo L., Wang J., Yun X., Chen Z. (2021). Fabrication of Aluminum–Magnesium Clad Composites by Continuous Extrusion. Mater. Sci. Eng. A.

[39] Kocich R., Szurman I., Kursa M., Fiala J. (2009). Investigation of Influence of Preparation and Heat Treatment on Deformation Behaviour of the Alloy NiTi after ECAE. Mater. Sci. Eng. A.

[40] Kocich R., Greger M., Macháčková A. (2010). Finite Element Investigation of Influence of Selected Factors on ECAP Process. Proceedings of the METAL 2010: 19th International Metallurgical and Materials Conference, Rožnov pod Radhoštěm, Czech Republic, 18–20 May 2010.

[41] Naizabekov A.B., Andreyachshenko V.A., Kocich R. (2013). Study of Deformation Behavior, Structure and Mechanical Properties of the AlSiMnFe Alloy during ECAP-PBP. Micron.

[42] Andreyachshenko V. (2019). Evolution of Al-Si-Mn-Fe Aluminum Alloy Microstructure in the Equal-Channel Angular Pressing with Back Pressure. Mater. Lett..

[43] Murashkin M., Medvedev A., Kazykhanov V., Krokhin A., Raab G., Enikeev N., Valiev R. (2015). Enhanced Mechanical Properties and Electrical Conductivity in Ultrafine-Grained Al 6101 Alloy Processed via ECAP-Conform. Metals.

[44] Derakhshan J.F., Parsa M.H., Jafarian H.R. (2019). Microstructure and Mechanical Properties Variations of Pure Aluminum Subjected to One Pass of ECAP-Conform Process. Mater. Sci. Eng. A.

[45] Kocich R., Kunčická L., Král P., Macháčková A. (2016). Sub-Structure and Mechanical Properties of Twist Channel Angular Pressed Aluminium. Mater. Charact..

[46] Kocich R., Kunčická L., Mihola M., Skotnicová K. (2013). Numerical and Experimental Analysis of Twist Channel Angular Pressing (TCAP) as a SPD Process. Mater. Sci. Eng. A.

[47] Kocich R., Fiala J., Szurman I., Macháčková A., Mihola M. (2011). Twist-Channel Angular Pressing: Effect of the Strain Path on Grain Refinement and Mechanical Properties of Copper. J. Mater. Sci..

[48] Kocich R., Macháčková A., Kunčická L. (2014). Twist Channel Multi-Angular Pressing (TCMAP) as a New SPD Process: Numerical and Experimental Study. Mater. Sci. Eng. A.

[49] Kocich R., Kunčická L., Macháčková A. (2014). Twist Channel Multi-Angular Pressing (TCMAP) as a Method for Increasing the Efficiency of SPD. IOP Conf. Ser. Mater. Sci. Eng..

[50] Kosinova A., Straumal B., Kilmametov A., Straumal P., Bulatov M., Rabkin E. (2020). Faceting of Twin Grain Boundaries in High-Purity Copper Subjected to High Pressure Torsion. Adv. Eng. Mater..

[51] Panfilov P., Tolmachev T.P., Pilyugin V.P., Chen Z., Zhang Z.L. (2021). On the Behavior of Rhenium under High-Pressure Torsion at Room Temperature. Mater. Lett..

[52] Panov D.O., Sokolovsky V.S., Stepanov N.D., Zherebtsov S.V., Panin P.V., Volokitina E.I., Nochovnaya N.A., Salishchev G.A. (2023). Effect of Interlamellar Spacing on Strength-Ductility Combination of β-Solidified γ-TiAl Based Alloy with Fully Lamellar Structure. Mater. Sci. Eng. A.

[53] Lotkov A., Kashin O., Grishkov V., Zhapova D., Krukovskii K., Gusarenko A., Girsova N., Bobrov D., Kashina O. (2022). Mechanical Properties of the Ti_49.8_Ni_50.2_ Alloy after Multi-Axial Forging at 573 K. Metals.

[54] Liu Y., Herrmann M., Schenck C., Kuhfuss B. (2019). Plastic Deformation Components in Mandrel Free Infeed Rotary Swaging of Tubes. Procedia Manuf..

[55] Zhang Q., Jin K., Mu D., Zhang Y., Li Y. (2015). Energy-Controlled Rotary Swaging Process for Tube Workpiece. Int. J. Adv. Manuf. Technol..

[56] Kocich R., Kursa M., Szurman I., Dlouhý A. (2011). The Influence of Imposed Strain on the Development of Microstructure and Transformation Characteristics of Ni–Ti Shape Memory Alloys. J. Alloys Compd..

[57] Kunčická L., Kocich R. (2022). Effect of Activated Slip Systems on Dynamic Recrystallization during Rotary Swaging of Electro-Conductive Al-Cu Composites. Mater. Lett..

[58] Barkov L.A., Mymrin S.A., Samodurova M.N., Dzhigun N.S., Latfulina Y.S. (2015). Compressibility of Tungsten and Molybdenum Bars during Rotary Swaging and Rolling. Russ. Metall. (Met.).

[59] Dyakonov G.S., Yakovleva T.V., Mironov S.Y., Stotskiy A.G., Modina I.M., Semenova I.P. (2023). Microstructure of the Advanced Titanium Alloy VT8M-1 Subjected to Rotary Swaging. Materials.

[60] Abdulstaar M.A., El-Danaf E.A., Waluyo N.S., Wagner L. (2013). Severe Plastic Deformation of Commercial Purity Aluminum by Rotary Swaging: Microstructure Evolution and Mechanical Properties. Mater. Sci. Eng. A.

[61] Chen X., Liu C., Jiang S., Chen Z., Wan Y. (2022). Fabrication of Nanocrystalline High-Strength Magnesium−Lithium Alloy by Rotary Swaging. Adv. Eng. Mater..

[62] Rogachev S.O., Sundeev R.V., Andreev V.A., Andreev N.V., Tabachkova N.Y., Korotkova N.O. (2022). The Microstructure and Conductivity of Copper–Aluminum Composites Prepared by Rotary Swaging. Phys. Met. Metallogr..

[63] Fan J., Sun Y., Yang H., Xie W., Tan Z., Chen G., Zhou Y., Zheng K., Xu J., Tan J. (2025). Influence of Ti Particles on Nanocrystallization and Mechanical Properties of Ti/Mg-Gd-Y-Zn-Zr Composites during Rotary Swaging. J. Alloys Compd..

[64] Kocich R., Kunčická L. (2023). Optimizing Structure and Properties of Al/Cu Laminated Conductors via Severe Shear Strain. J. Alloys Compd..

[65] Wang H., Han J., Hao Q. (2015). Fabrication of Laminated-Metal Composite Tubes by Multi-Billet Rotary Swaging Technique. Int. J. Adv. Manuf. Technol..

[66] Panov D., Kudryavtsev E., Naumov S., Klimenko D., Chernichenko R., Mirontsov V., Stepanov N., Zherebtsov S., Salishchev G., Pertcev A. (2023). Gradient Microstructure and Texture Formation in a Metastable Austenitic Stainless Steel during Cold Rotary Swaging. Materials.

[67] Panov D.O., Kudryavtsev E.A., Chernichenko R.S., Naumov S.V., Sekhar K.C., Stepanov N.D., Zherebtsov S.V., Salishchev G.A., Pertsev A.S. (2025). Significantly Enhanced Mechanical Properties of Metastable Austenitic Stainless Steel with Large-Scale Gradient Structure. Mater. Sci. Eng. A.

[68] Volodko S., Markova G., Yudin S., Permyakova D., Alimov I., Evstratov E., Moskovskikh D., Khort A., Kasimtsev A. (2024). Torsional Behavior of Ni-Rich NiTi Alloys Obtained by Powder Metallurgy and Hot Deformation. Sci. Rep..

[69] Shuytcev A., Markova G., Kasimtcev A., Volod’ko S. (2017). The Influence of Deformation on the Structure and Properties of TiNi Sintered Powder. Mater. Today Proc..

[70] Lee S.K., Yun S.H., Joo H.G.G., Noh S.J. (2014). Deuterium Transport and Isotope Effects in Type 316L Stainless Steel at High Temperatures for Nuclear Fusion and Nuclear Hydrogen Technology Applications. Curr. Appl. Phys..

[71] Macháčková A., Kocich R., Bojko M., Kunčická L., Polko K. (2018). Numerical and Experimental Investigation of Flue Gases Heat Recovery via Condensing Heat Exchanger. Int. J. Heat Mass Transf..

[72] Lin K., Qiao J., Gu D., Wang H., Shi B., Zhang W., Shan J., Xu Y., Tian L. (2023). Active Screen Plasma Nitriding of Laser Powder Bed Fusion Processed 316L Stainless Steel for the Application of Fuel Cell Bipolar Plates. Virtual Phys. Prototyp..

[73] Romanovski V., Frantskevich V., Kazlouski V., Kasach A., Paspelau A., Hedberg Y., Romanovskaia E. (2020). Inappropriate Cleaning Treatments of Stainless Steel AISI 316L Caused a Corrosion Failure of a Liquid Transporter Truck. Eng. Fail. Anal..

[74] Avanzini A. (2022). Fatigue Behavior of Additively Manufactured Stainless Steel 316L. Materials.

[75] de Moraes F.P., Alves S.F., Plaut R.L., Padilha A.F. (2019). Degradation of Microstructure and Properties of an AISI 316L Steel Pipe after More than 100,000 Hours Usage at 640 °C in a Petrochemical Industry. Procedia Struct. Integr..

[76] Zach L., Kunčická L., Růžička P., Kocich R. (2014). Design, Analysis and Verification of a Knee Joint Oncological Prosthesis Finite Element Model. Comput. Biol. Med..

[77] Grenadyorov A.S., Oskirko V.O., Solovyev A.A., Oskomov K.V., Khlusov I.A. (2021). Wear and Corrosion Resistance of A-C:H:SiO_x_ Coating on Medical 316L Stainless Steel. J. Mater. Eng. Perform..

[78] Ferrandini P.L., Rios C.T., Dutra A.T., Jaime M.A., Mei P.R., Caram R. (2006). Solute Segregation and Microstructure of Directionally Solidified Austenitic Stainless Steel. Mater. Sci. Eng. A.

[79] Choi J., Seok C.S., Park S., Kim G. (2019). Effect of High-Temperature Degradation on Microstructure Evolution and Mechanical Properties of Austenitic Heat-Resistant Steel. J. Mater. Res. Technol..

[80] Aydoğdu G.H., Aydinol M.K. (2006). Determination of Susceptibility to Intergranular Corrosion and Electrochemical Reactivation Behaviour of AISI 316L Type Stainless Steel. Corros. Sci..

[81] Hochstrasser-Kurz S., Gümpel P., Arlt N. (2008). Sensitization Behaviour of Manganese-Alloyed Austenitic Stainless Steels. Steel Res. Int..

[82] Jones R., Randle V. (2010). Sensitisation Behaviour of Grain Boundary Engineered Austenitic Stainless Steel. Mater. Sci. Eng. A.

[83] Saboori A., Aversa A., Marchese G., Biamino S., Lombardi M., Fino P. (2020). Microstructure and Mechanical Properties of AISI 316L Produced by Directed Energy Deposition-Based Additive Manufacturing: A Review. Appl. Sci..

[84] Zanca C., Milazzo A., Campora S., Capuana E., Pavia F.C., Patella B., Lopresti F., Brucato V., La Carrubba V., Inguanta R. (2023). Galvanic Deposition of Calcium Phosphate/Bioglass Composite Coating on AISI 316L. Coatings.

[85] Majumdar J.D.D., Kumar A., Pityana S., Manna I. (2018). Laser Surface Melting of AISI 316L Stainless Steel for Bio-Implant Application. Proc. Natl. Acad. Sci. India Sect. A Phys. Sci..

[86] Pragana J.P., Pombinha P., Duarte V.R., Rodrigues T.A., Oliveira J.P., Bragança I.M., Santos T.G., Miranda R.M., Coutinho L., Silva C.M. (2020). Influence of Processing Parameters on the Density of 316L Stainless Steel Parts Manufactured through Laser Powder Bed Fusion. Proc. Inst. Mech. Eng. B J. Eng. Manuf..

[87] Bedmar J., Riquelme A., Rodrigo P., Torres B., Rams J. (2021). Comparison of Different Additive Manufacturing Methods for 316L Stainless Steel. Materials.

[88] Bakhtiarian M., Omidvar H., Mashhuriazar A., Sajuri Z., Gur C.H. (2024). The Effects of SLM Process Parameters on the Relative Density and Hardness of Austenitic Stainless Steel 316L. J. Mater. Res. Technol..

[89] Crisafulli D., Fintová S., Santonocito D., D’Andrea D. (2024). Microstructural Characterization and Mechanical Behaviour of Laser Powder Bed Fusion Stainless Steel 316L. Theor. Appl. Fract. Mech..

[90] Chepkoech M., Owolabi G., Warner G. (2024). Investigation of Microstructures and Tensile Properties of 316L Stainless Steel Fabricated via Laser Powder Bed Fusion. Materials.

[91] Gu J., Li R., Qiu Y., Yue H., Liu B., Gu H. (2020). Microstructure, Mechanical Properties, and Residual Stress Distribution of AISI 316L Stainless Steel Part Fabricated by Laser Metal Deposition. Scanning.

[92] Bemani M., Parareda S., Casellas D., Mateo A., Das R., Molotnikov A. (2024). Rapid Fatigue Evaluation of Additive Manufactured Specimens: Application to Stainless Steel AISI 316L Obtained by Laser Metal Powder Bed Fusion. Int. J. Fatigue.

[93] Ding H., Zou B., Wang X., Liu J., Li L. (2023). Microstructure, Mechanical Properties and Machinability of 316L Stainless Steel Fabricated by Direct Energy Deposition. Int. J. Mech. Sci..

[94] Portella Q., Chemkhi M., Retraint D. (2020). Influence of Surface Mechanical Attrition Treatment (SMAT) Post-Treatment on Microstructural, Mechanical and Tensile Behaviour of Additive Manufactured AISI 316L. Mater. Charact..

[95] Hlaváč L.M., Kocich R., Kunčická L., Hlaváčová I.M., Gřunděl J. (2025). Effect of Thermomechanical Post-Processing of Additively Manufactured AISI 316L Steel on Abrasive Water Jet Wear. Wear.

[96] Bunchoo N., Wongpinkaew K., Kukiatkulchai E., Kaewkumsai S., Viyanit E. (2022). Effects of Thermal History on Sensitization Behavior and Charpy Impact Property of Type 316L and 316 Stainless Steels for Applications in a Fired Heater. Eng. Fail. Anal..

[97] Kiahosseini S.R., Mohammadi Baygi S.J., Khalaj G., Khoshakhlagh A., Samadipour R. (2018). A Study on Structural, Corrosion, and Sensitization Behavior of Ultrafine and Coarse Grain 316 Stainless Steel Processed by Multiaxial Forging and Heat Treatment. J. Mater. Eng. Perform..

[98] Elangeswaran C., Cutolo A., Muralidharan G.K., de Formanoir C., Berto F., Vanmeensel K., Van Hooreweder B. (2019). Effect of Post-Treatments on the Fatigue Behaviour of 316L Stainless Steel Manufactured by Laser Powder Bed Fusion. Int. J. Fatigue.

[99] Salman O.O., Gammer C., Chaubey A.K., Eckert J., Scudino S. (2019). Effect of Heat Treatment on Microstructure and Mechanical Properties of 316L Steel Synthesized by Selective Laser Melting. Mater. Sci. Eng. A.

[100] Beausir B., Fundenberger J.J. (2017). ATEX Software—Analysis Tools for Electron and X-Ray Diffraction. http://www.atex-software.eu.

[101] Krakhmalev P., Fredriksson G., Thuvander M., Åsberg M., Vilardell A.M., Oikonomou C., Maistro G., Medvedeva A., Kazantseva N. (2020). Influence of Heat Treatment under Hot Isostatic Pressing (HIP) on Microstructure of Intermetallic-Reinforced Tool Steel Manufactured by Laser Powder Bed Fusion. Mater. Sci. Eng. A.

[102] Pelevin I.A., Ozherelkov D.Y., Nalivaiko A.Y., Bodyakova A.I., Chernyshikhin S.V., Zotov B.O., Korshunov A.V., Gromov A.A. (2022). AlSi_10_Mg/AlN Interface Grain Structure after Laser Powder Bed Fusion. Metals.

[103] Semikolenov A., Kuznetsov P., Bobkova T., Shalnova S., Klimova-Korsmik O., Klinkov V., Kobykhno I., Larionova T., Tolochko O. (2021). Microstructure Evolution of FeNiCoCrAl_1.3_Mo_0.5_ High Entropy Alloy during Powder Preparation, Laser Powder Bed Fusion, and Microplasma Spraying. Materials.

[104] Khan M., Shahriari D., Jahazi M., Morin J.B. (2021). Interactions Between Dynamic Softening and Strengthening Mechanisms During Hot Forging of a High-Strength Steel. Front. Mech. Eng..

[105] Humphreys F.J., Hetherly M., Rollett A., Rohrer G.S. (2004). Recrystallization and Related Annealing Phenomena.

[106] Verlinden B., Driver J., Samajdar I., Doherty R.D. (2007). Thermo-Mechanical Processing of Metallic Materials.

[107] Simpson G., Grant J., Weihs T.P., Ramesh K.T. (2025). Size-Dependent Fragment Shape in High-Velocity Anvil Impact of Spherical Metal Powder-Compacts. Acta Mater..

[108] Canelo-Yubero D., Kocich R., Šaroun J., Strunz P. (2023). Residual Stress Distribution in a Copper-Aluminum Multifilament Composite Fabricated by Rotary Swaging. Materials.

[109] Otto F., Frenzel J., Eggeler G. (2011). On the Evolution of Microstructure in Oxygen-Free High Conductivity Copper during Thermo-Mechanical Processing Using Rotary Swaging. Int. J. Mater. Res..

[110] Kunčická L., Kocich R. (2018). Deformation Behaviour of Cu-Al Clad Composites Produced by Rotary Swaging. IOP Conf. Ser. Mater. Sci. Eng..

[111] Kocich R., Kunčická L., Minárik P. (2025). Crossing the Limits of 316L Steel Fabricated by Powder Bed Fusion by Thermomechanical Post-Processing. Mater. Des..

[112] Talonen J., Hänninen H. (2007). Formation of Shear Bands and Strain-Induced Martensite during Plastic Deformation of Metastable Austenitic Stainless Steels. Acta Mater..

